# Knowledge Domain and Emerging Trends in Wound, Ostomy, and Continence Nursing: A Scientometric Review Based on CiteSpace and VOSviewer Analysis

**DOI:** 10.1155/nrp/8836038

**Published:** 2025-09-17

**Authors:** Qiaoling Li, Yuejuan Zhang, Wenli Zhao, Jiabin Li, Xiaoqian Wang, Yan Tang

**Affiliations:** ^1^School of Nursing, Hunan University of Chinese Medicine, Changsha, China; ^2^Department of Nursing, The First Affiliated Hospital of Hunan University of Chinese Medicine, Changsha, China; ^3^Dermatology Department, The Second Affiliated Hospital of Hunan University of Chinese Medicine, Changsha, China

## Abstract

**Aim:** The aim of this study is to explore the international landscape and hot topics within the field of wound, ostomy, and continence (WOC) nursing over recent years.

**Methods:** Literature on WOC nursing published between 2012 and 2024 was retrieved from the Web of Science database. VOSviewer was employed as the primary tool, complemented by CiteSpace, to conduct a bibliometric analysis. This included examining collaborative networks at the author, institutional, and national levels, alongside keyword visualizations employing clustering, time zone diagram analysis, and emergence analyses.

**Results:** An initial search identified 3474 publications. These were subsequently grouped into eight central clusters, encompassing the work of 6341 authors. Analysis revealed current research hotspots, including the field of pressure injury, ostomy management, wound management, incontinence, skin damage, therapy technology, nursing care, and telehealth.

**Conclusion:** First, scientific research attention in the field of WOC nursing is not high. Second, the uneven development of WOC nursing is mainly reflected in the imbalance in the number of publications between journals and countries. Third, the research focus of WOC nursing is in the wound care sector, among which pressure injuries have always been a research hotspot.

## 1. Introduction

In recent years, with the intensification of population aging and the rising incidence of traumatic events, the number of patients with chronic wounds, artificial stomas, and incontinence-associated dermatitis has gradually increased [1, 2], posing new demands on global wound, ostomy, and continence (WOC) nurses [3, 4]. WOC specialty nursing is an advanced practice domain dedicated to the management of complex wound healing disorders, ostomy-related conditions, and urinary/fecal incontinence [5]. A wound, ostomy, and continence nurse (WOCN) is a registered nurse (RN) who formally trained WOC specialist (either full scope or discipline specific) and holds at least a bachelor's degree. These specialized nurses are trained to manage wound care, ostomy-related complications, and incontinence, while also providing psychological support, rehabilitation guidance, and disease-specific education to patients and their families, particularly those with gastrointestinal, genitourinary, or anorectal disorders [6, 7]. In developed nations (e.g., the United States), the framework for WOC nurse training and management is highly developed, encompassing standardization in candidate selection, educational content, certification, and position structuring [8, 9]. China has witnessed a significant expansion of its professional nursing workforce in recent years [10]. Nevertheless, a discernible disparity persists when compared to developed nations, highlighting the need to adopt international benchmarks in this domain.

In order to align with global standards in the WOC nursing field and to develop WOC nursing more rapidly and effectively, it is necessary to grasp the changing dynamics and future directions in the international WOC nursing field from multiple levels and perspectives in recent years. Bibliometrics, a quantitative analysis method grounded in mathematical statistics, enables systematic organization of extensive textual data [11, 12]. It facilitates the tracing of developmental trajectories and the identification of emerging research trends [13]. At present, lots of bibliometric analysis software have been developed for drawing scientific knowledge maps, such as Bibliometrix, CiteSpace, VOSviewer, Sci2, the Network Workbench (NWB), Pajek, and Metaknowledge, which are suitable for topic analysis and network analysis. VOSviewer and CiteSpace are employed to conduct quantitative analysis on research entities including literature, keywords, authors, and collaborative institutions or countries, employing quantitative, statistical methodologies. Furthermore, VOSviewer outperforms other bibliometric analysis tools in terms of its visualization functionality. In addition, an important feature of CiteSpace is the ability to perform keyword time zone analysis. This feature enhances the direct observation of changes in popular trends. Therefore, this study employs VOSviewer and CiteSpace to classify the knowledge domains within the field of WOC nursing research, aiming to better understand the global development of the WOC nursing profession, summarize and analyze the current state of WOC nursing development, and promote the continuous and positive advancement of the WOC nursing specialty.

## 2. Data and Methods

### 2.1. Data Collection

To explore the latest research trends in WOC nursing, we used the Web of Science to retrieve WOC nursing-related studies published up to June 2024. The index term included “Wound, Ostomy, and Continence Nursing” OR “WOC Nursing.” Thus, 3474 studies were found. The resulting dataset was utilized for subsequent processing and visualization in CiteSpace and VOSviewer. Studies were downloaded on June 6, 2024. The exported data consisted of full-recorded and referenced references, with 500 plain text files saved per group.

### 2.2. Data Analysis

The literature data retrieved from Web of Science were saved in the input folder and subsequently analyzed using CiteSpace and VOSviewer.

On the CiteSpace software, the data were first deduplicated by “Data Processing Utilities”: select the input folder for input, and the newly created date folder for output. Then, the date folder and the newly created project folder were imported, thus further data analysis could be proceed. Running CiteSpace 6.2.R2, the data time, was set from January 2012 to July 2024, year per slice was set to 1, and node types were set to “keyword,” and other default options. The size of the node in the graph represents the frequency of occurrence, the connection represents the co-occurrence relationship, and the centrality is an indicator of the importance of the node. Its value ≥ 0.1 indicates that the node plays an important role in the knowledge graph [14].

On the VOSviewer software, the co-occurrence analysis thresholds for keywords, authors, research institutions, and countries were configured as follows.

1. Keyword setting: In VOSviewer, a minimum occurrence threshold of 5 was applied to the keywords. Out of 3299 keywords, 309 met this criterion.
2. Author co-occurrence: In this study, the VOSviewer software parameter settings were as follows: the counting method was fractional counting, ignoring the literature research with more than 25 authors and setting the “minimum number of authors per documents of an author” (the minimum number of documents per author) to 8 in 6341 authors, and a total of 113 authors reached the threshold.
3. Co-occurrence of research institutions: A minimum publication threshold of 6 documents per organization was applied. Among 2498 research institutions, 130 met this criterion.
4. Co-occurrence of countries: A minimum publication threshold of 5 documents was set per country. Of the 55 countries included, 27 met this criterion.

## 3. Results

### 3.1. Publication Years and Journals

**Figure 1:**
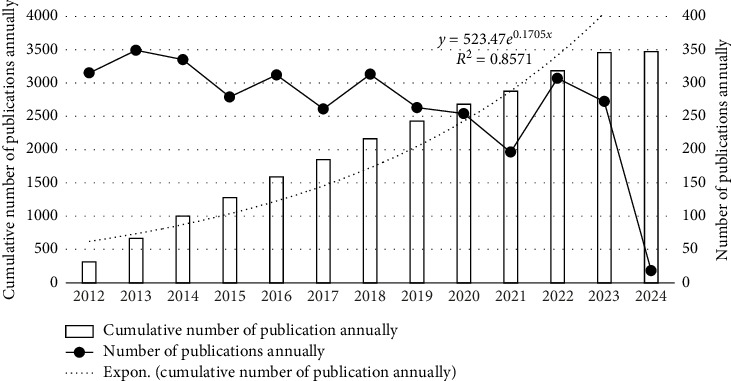
Time sequence of relevant papers on WOC nursing published from 2012 to 2024 in Web of Science.

In 1992, “Enterostomal Therapist (ET)” was formally changed to “Wound, Ostomy, and Continence Nurse (WOCN)” [15]. In 2010, the American Nursing Association (ANA) formally defined ET-led WOC nursing as “the specialty that provides wound ostomy continence care and standards of practice” [5]. This designation marked the transition of WOC nursing from a professional nursing focus to a recognized nursing specialty, which fully reflects the importance and inevitability of WOC nursing specialization in clinical nursing work. An analysis of publication trends in the SCI database reveals substantial scholarly activity in WOC nursing. Since 2012, a total of 3474 WOC-related publications have been indexed. Publication numbers demonstrated an overall declining trend with periodic fluctuations through 2021. The peak annual output occurred in 2013 with 349 publications, while 2021 saw the lowest output at just 196 articles. A regression analysis of cumulative publications yielded a strong fit (*R*^2^ = 0.8571), as illustrated in Figure 1.

Based on the collected 3474 publications, the journal sources were identified using the Web of Science database, and the top 13 journals are presented in Table 1.

Journal of Wound, Ostomy, and Continence Nursing (JWOCN) ranked first (3320 articles) for published articles on WOC nursing in hundreds of journals. JWOCN is the official journal of the WOCN society and is the premier publication for WOC practice and research. The Journal's mission is to disseminate cutting-edge evidence and pioneering research that informs and advances specialized healthcare practices. Since its establishment in 1994, the journal has published a total of 3082 articles, with an H-index of 45, an annual publication of 904 articles, and a self-citation rate of 43.7%. The second-ranked journal was Ostomy Wound Management (JOWM), with 18 WOC nursing-related articles. JOWM was founded in March of 1980 as “Ostomy Management.” In 1985, JOWM significantly broadened its scope to encompass the interdisciplinary nature of WOC nursing, incorporating related aspects of dermatology and nutritional science. This expansion successfully increased both its content diversity and professional readership base. While JOWM has not yet been included in the Journal Citation Reports (JCR). It is noteworthy that almost all of the top 10 journals by publication volume belong to WOC nursing specialty journals, with relatively limited participation from other non-WOC nursing journals. This illustrates that the scientific research on WOC nursing needs to be strengthened.

### 3.2. Keywords Heat and Cluster Analysis

Keywords are used to express/represent the subject content of literatures, which brings great convenience to the storage and retrieval of literatures. Keywords play an important role in understanding the general situation of the research field, which is a comprehensive and high-level way identify research hotspots and emerging trends within the field [16]. The frequency of a keyword's occurrence reflects the total number of scholarly publications associated with that term. A higher keyword frequency indicates a greater volume of related research and a more focused, in-depth investigation within that specific area [13]. In terms of the keyword heat and cluster analysis, VOSviewer was used for clustering and heat analysis; CiteSpace was used to analyze the time zone map and keyword emergence.

#### 3.2.1. Cluster Analysis of Keywords

**Figure 2:**
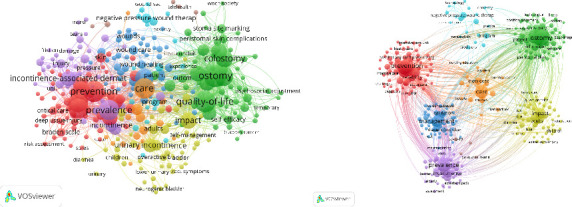
Keywords clustering map of WOC nursing from 2012 to 2024: (a) unadjusted and (b) after adjustment.

The literature obtained from the Web of Science database was processed using VOSviewer Version 1.6.19. Following the removal of duplicate and irrelevant keywords, cluster analysis was conducted on the keyword set, with a specific focus on examining high-frequency terms through hotspot analysis. The software was used to generate the visual density map and cluster map.  Figure 2(a) presents the initial keyword clustering result. To improve clarity, the network was refined using Pajek for layout adjustment and then reimported into VOSviewer, resulting in a more distinct and unified cluster map shown in Figure 2(b).

As illustrated in Figure 2(b), the keywords are categorized into eight distinct clusters. The primary research themes can be summarized into the following eight categories: (1) prevention, risk factors, skin breakdown, acute care, intensive care, pressure injury (red); (2) ostomy, colorectal cancer, colostomy, peristomal complication, quality of life, self-care, adaption (green); (3) management, wounds care, diabetic food, venous leg ulcers (dark blue); (4) urinary, urinary incontinence, urge incontinence (yellow); (5) pressure, moisture-associated skin damage, barrier, barrier function, skin care (purple); (6) negative pressure wound therapy, technology (light blue); (7) care, nursing care, knowledge, nursing education (orange); and (8) telehealth, telemedicine (brown).

The results of cluster analysis show that the research hotspots of environmental WOC nursing in the world from 2012 to 2024 mainly focus on prevention of pressure injure, ostomy management, wound management, incontinence, skin damage, therapy technology, nursing care, and telehealth. Based on the keyword dataset extracted through VOSviewer, the 12 most frequently occurring keywords are presented in Table 2. “Word Frequency” represents the total frequency of each keyword, with a higher value indicating greater popularity of the keyword within the research topic. “Total Correlation Strength” denotes the total correlation strength of a keyword, which refers to the overall correlation between the keyword and the other keywords in the topic. A higher value indicates a stronger connection between the keyword and the other keywords in the research topic.

**Figure 3:**
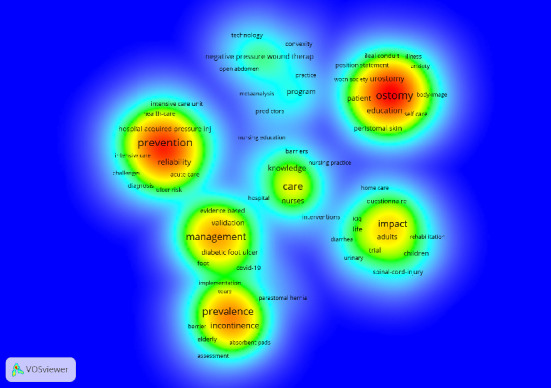
Keyword density visualization.

Based on the density visualization diagram (Figure 3), the research hotspots in WOC nursing were identified. In the resulting visualization, darker shades indicate areas of greater research activity and focus [13]. Our study shows that there are many hot spots in the prevention of pressure injure, ostomy management, wound management, incontinence, and skin damage. It shows that the management of WOC has attracted the attention and research of most scholars.

#### 3.2.2. Time Zone Diagram and Keyword Emergence Analysis

Using CiteSpace, the keyword time zone map and keyword emergence graph were generated. These visualizations facilitate the identification of temporal patterns in keyword appearance, thereby aiding in the recognition of research hotspots and the forecasting of future trends.

##### 3.2.2.1. Time Zone Diagram Analysis

**Figure 4:**
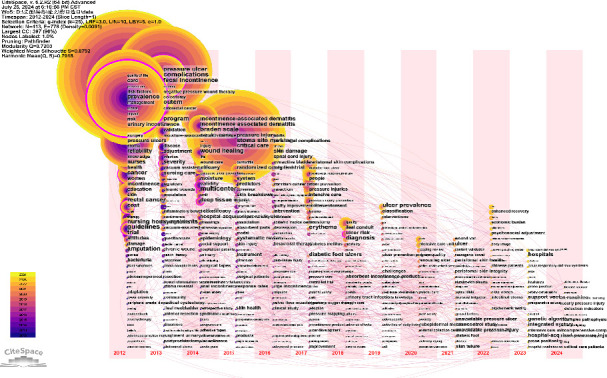
Time zone map of WOC nursing keywords.

With CiteSpace, the keyword time zone map was drawn and modified (Figure 4). The layout positions smaller circles at the base and larger circles toward the top, arranged in an organized manner. This configuration offers an intuitive visualization of the temporal distribution of WOC nursing keywords across international time zones. The larger the circle, the more times it is cited, and there are more discussions and research. Meanwhile, the size of the words is set by degree, the larger the words, the high degree the keyword term. Beneath the visualization, a corresponding timeline is displayed. Using a 1-year time slice, the temporal distribution of each research topic can be clearly observed. As can be seen from Figure 4, the most studied keyword was “quality of life” that appeared in 2012, and the keywords that appeared in 2012 were also the broadest and most comprehensive. Although the volume of discussions and research on WOC nursing varied annually from 2012 to 2017, the topic maintained relatively strong popularity, with a stable number of newly emerging keywords each year. After 2017, the number of keywords that appeared for the first time every year showed a significant decrease compared with previous years, and the amount of discussion and research was also less. It is worth noting that wound management has always been a hot topic in WOC nursing. Almost every year, the keywords that appear for the first time included wound management content, and wound management content account for a large proportion of nodes with strong betweenness centrality (purple circle keywords in Figure 4).

##### 3.2.2.2. Keyword Emergence Analysis

From the literature on WOC nursing, it can be seen that the word “woman” has appeared since 2012, which may be due to the high association of incontinence with women, and can be understood as the representative keyword of continence in 2012. Since ET-led WOC nursing was officially defined in 2010, as of 2016, the word “woman” has received 4.69 correlation intensity. It shows that from 2012 to 2016, continence management has been attracted the attention and exploration of relevant scholars. At the same time, the keywords that emerged in 2012 was “pressure ulcers,” which can be understood the concept of pressure injuries during this period. Besides, the key words “pressure,” “negative pressure wound therapy,” and “vacuum assisted closure” that appeared in 2013, including “pressure ulcer prevention” that appeared in 2014, all related to pressure ulcers. In total, all words above have high association with pressure injure and is also a hot topic in wound management. In 2016, the American Pressure Injury Advisory Committee issued a statement renaming pressure ulcers as pressure injuries, and the management of pressure ulcers entered a new stage. As can be seen from Table 3, the keywords related to pressure ulcers continued until around 2015. The term “pressure injury” emerged in 2015 and became a hot topic in wound management from 2017 to 2020. It is worth noting that the term “incontinence-associated dermatitis,” which appeared in 2014, involving wound management and continence management two aspects, further illustrating the important role WOC nursing plays in a holistic approach to WOC care.

From the original ET to WOC, ostomy management has always been the basis of WOC nursing. However, with the development of medicine, the intersection of disciplines becomes deeper and deeper, and the scope of WOC nursing becomes wider and wider. The focus of WOCN has also shifted from ostomy management to more complex wound management and holistic care. It can be seen from the keyword emergence that keywords related to ostomy management account for a small proportion, among which only “ulcers,” “ulcer prevention,” and “ulcer risk” may be related to ostomy management.

### 3.3. Cluster Analysis of Author, Research Institutions, and Countries

#### 3.3.1. Cooperation Among Authors

**Figure 5:**
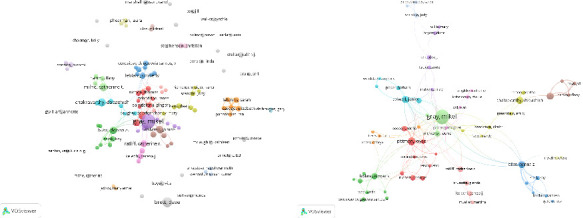
(a) Co-occurrence graph of author collaboration and (b) co-occurrence diagram of author collaboration (small group of 63 people).

Through the operational data, 6341 authors who published WOC nursing-related literature were obtained in VOSviewer. The presence of leading scholars is pivotal to the advancement of a research field, as they serve as the primary drivers of its development and innovation [13]. Gray, Mikel (119 papers), as the main contributors in the field of WOC nursing, collaborated closely with Mcnichol, Laurie (25 papers). According to Price's law [17], if the formula for core authors is Nmin = 0.749 × √Nmax, then Nmin ≈ 8.16, that is, the authors who have published 8 or more papers are the core authors of WOC nursing, accounting for 1.58% (100 in total). When the total number of papers published by core authors reaches 50% of all the papers in a given field, it signifies the establishment of a stable core author group. In this study, core authors accounted for 45% (1562 papers) of total publications, which is below the 50% threshold. Therefore, it demonstrates that a core author group has not yet been established in the field of WOC nursing. The network of authors is shown in Figure 5. Some of the 113 items in the network are not connected to each other (as shown in Figure 5(a)). The largest connection consisted of 63 projects (shown in Figure 5(b)), showing 63 authors in 13 collaborative groups, with the largest number of group members is nine. There are four members in the four groups, six, five, three members in every two groups, and eight and two members in the other two groups.

WOC nursing mainly includes wound management, ostomy management, and continence management, which has led to more detailed research directions, such as pressure injury, diabetic foot, new dressings and management devices. The training criterion of WOC is to provide WOC care. Senior WOCN's research directions are involved in almost all three aspects, and most of these authors are closely connected. However, the new dressings and management devices belong to materials science. Although WOCN are very involved in new dressings and management devices such as NPWT, but there are few direct links between the two groups. Only a few scholars have established links and cooperation between the members of the groups through disease management. Among them, Gray Mikel, who has published the most papers, is involved in all three research directions, but his specialty is continence. Brett Dave, as the second author of the post, whose subject categories are “nursing” and “Food Science & Technology,” mainly focus on new dressings and management devices. Pittman Joyce, posted third, is also involved three directions all, but the focus is on the care of pressure injuries. Therefore, there is direct link between Gray Mikel and Pittman Joyce in the collaborator network, while Brett Dave has no links with the first two authors. It is noteworthy that Mikal Grey, as the editor of JWOCN, may have an impact on the publication frequency. Information of the authors with more than 20 articles is shown in Table 4.

#### 3.3.2. Cooperation Among International Research Institutions

**Figure 6:**
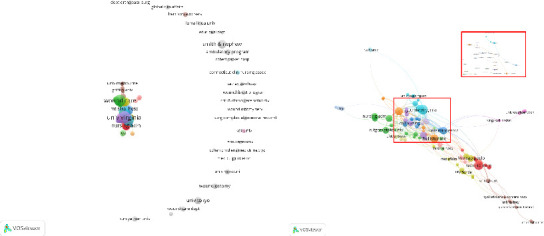
(a) Co-occurrence graph of research institutions collaboration and (b) co-occurrence diagram of research institutions collaboration (small group of 99 research institutions).

Scientific project collaboration and joint research are vital approaches to advancing scientific development, facilitating knowledge exchange, and enhancing efficiency and resource utilization, while promoting the application and transformation of research outcomes. The output of WOC nursing among international research institutions from 2012 to 2024 is shown in Figure 6. The cooperation between scientific research institutions is not close, with many scientific research institutions having little to no contact with others. But some of them form a close network of cooperation (as shown in Figure 6(a)). According to the connection between institutions, the University of Virginia (UVA) has established the closest cooperative relationships with Duke University and the University of Minnesota System, respectively. At the same time, the University of Chicago and Hollister Incorporated also cooperate most closely, as shown in Figure 6(b). Research institutions should actively explore and promote collaboration and joint research on scientific projects to make greater contributions to technological innovation and social development.

The visual analysis results of interagency cooperation obtained by VOSviewer software are shown in Table 5. UVA has published 61 papers, ranking first. Other highly productive institutions with more than 20 publications are Hollister Incorporated (43), University of Chicago (42), University of Minnesota (37), The Medical University of South Carolina (35), Smith & Nephew (30), University of São Paulo (30), Rutgers, The State University of New Jersey (29), University of Pittsburgh (29), The University of Tokyo (22), and Duke University (21). Among the 11 institutions that published more than 20 papers from 2012 to 2024, the United States accounts for the largest proportion, reflecting that American research institutions are increasingly active in WOC nursing. In addition, it also includes two medical technology companies, Hollister Incorporated and Smith & Nephew, both of which are dedicated to providing medical products for wound care, ostomy care, and continence care. Although they have also made significant contributions to the development of WOC care, these companies have commercial interests in the fields of wound and ostomy pouch care. The presence of industry-affiliated entities may lead to corresponding biases in published research.

The results shown in Table 6 were obtained by ranking all institutions according to the number of citations. The most cited is UVA. UVA is one of the most comprehensive institutions of higher education in the United States. The top five institutions were UVA, the University of Chicago, the University of Pennsylvania, Hollister Incorporated, and the University of Minnesota. Eight of the 12 institutions with more than 20 publications also rank among the top 15 in terms of citations. American scientific research institutes publish more papers, accounting for 72.7% of the top 12, and 8 institutions are all among the top 15 cited. The results show that relevant scientific research institutions in the United States have a relatively authoritative position in WOC nursing. The reason may be that the development of WOC nursing in the United States started early, with a relatively mature training and management system for specialized WOC nurses, and standardized related systems, which have promoted the development of institutions and research in the field of WOC nursing.

#### 3.3.3. Cooperation Between International Countries

**Figure 7:**
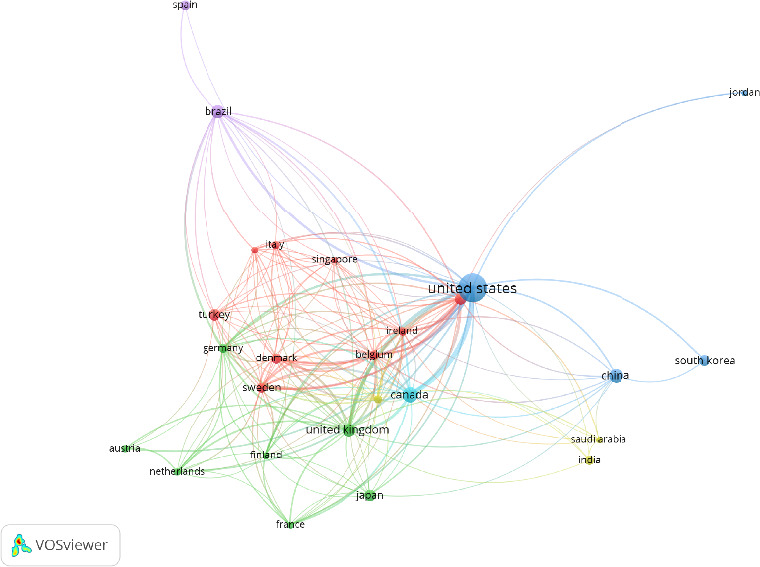
Cooperation among WOC nursing countries.

In the analysis of knowledge domains within WOC nursing, the country is employed as the unit of analysis [18]. In VOSviewer, select “Country” as the research node to obtain the knowledge map of national (regional) cooperation. The results shown in Figure 7, there are 55 nodes, and one country (region) is represented by a node. The size of the node reflects the number of documents issued by different countries. The largest node in the figure is the United States in blue, indicating that the United States dominates the thematic area, which also corroborates the previously mentioned dominant position of relevant U.S. research institutions in WOC nursing. This is followed by Canada, China, and the United Kingdom, and the number of publications is also higher. According to the thickness of the connection between countries, the United States and Canada have the closest cooperative relationship, followed by the United States and the United Kingdom.

**Figure 8:**
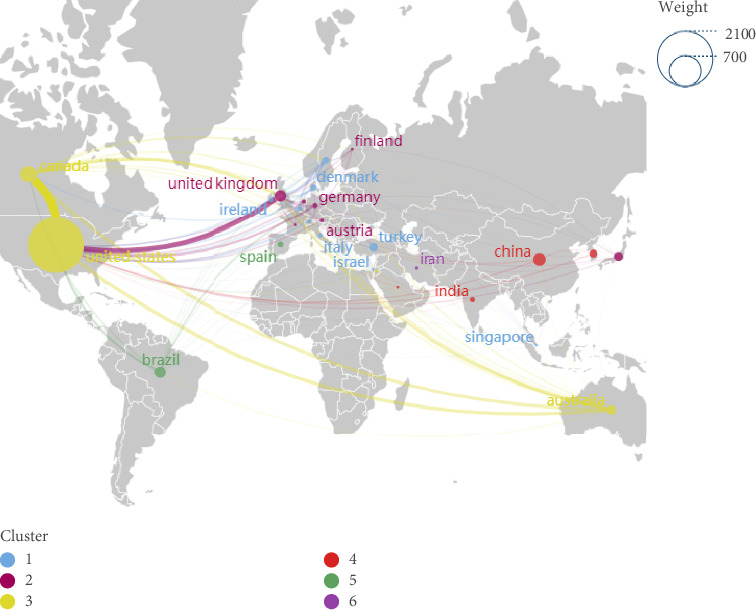
Cooperation among WOC nursing countries on the world map.

Figure 7 obtained by VOSviewer was exported in GML format and the running was performed in Scimago. As shown in Figure 8, the diagram annotates countries according to their geographic distribution on the world map. The size of each country's circle represents the volume of published literature, with larger circles indicating more publications. The thickness of the connecting lines between countries represents the degree of cooperation between them, with thicker lines indicating closer collaboration. The results show that the field of WOC nursing mainly covers the Eurasian continent, and although there are cooperative relationships in scientific research within each continent, the linkages are relatively weak. Cooperation between continents are also largely maintained by a few countries with a large number of publications, indicating that the popularity of WOC nursing is not high enough and the development is uneven across countries.

According to the issuance of documents, the ranking of WOC nursing is shown in Table 7. The United States ranks first in publication, citation frequency, and total link strength; Canada ranks second in publication and total link strength but is slightly lower than the United Kingdom in terms of citations; China ranks third in terms of publication, but it has a low number of citations and total links, indicating that the relationship between China and other countries in terms of scientific research cooperation is not very close. Analyzing the reasons, it may be related to language barriers. Additionally, the late start of WOC nursing in China, the limited author collaboration network, and the differences in the focus of nursing research could all contribute to the imbalance in China's publication output and scientific research collaboration. Furthermore, the lack of close cooperation between clinical nurses with rich clinical experience but weaker research capabilities and research nurses with strong research capabilities but insufficient clinical experience, leading to a separation between clinical practice and research, could also be a reason for this imbalance.

## 4. Strengths and Limitations

Our study explored the research progress of WOC nursing using literature in this field through quantitative scientific measurement methods. The study results visually demonstrate the overall trends and research hotspots of WOC nursing and can serve as a reference for researchers interested in conducting more in-depth studies in this field. However, our study still has the following limitations: (1) we only collected articles from a single database (i.e., the WoS database) and may have missed some data about this field in other databases; (2) the search terms used a single combination of words (i.e., WOC nursing), which may miss some data that only use one of the words wound, ostomy, or continence as a keyword (we merged the search results of single keywords, but the number of documents was reduced); and (3) data analysis software restrictions prevent data from different databases from being combined for analysis. This study did not conduct a search of Chinese databases and only analyzed information from one English database, so the content may not be comprehensive enough. In summary, due to the limitation of the software's inability to perform multidatabase data consolidation analysis, the research findings are derived solely from a single English database, which may introduce data selection bias and potentially lack comprehensive global representation. In addition, Mikal Grey's editorial role may influence the publication frequency, and the direct commercial interests in WOC nursing products (e.g., dressings and ostomy appliances) of some companies and their dominant representation in bibliometric data can distort the perceived landscape of scientific contribution.

## 5. Conclusions

1. Bibliometric results show that WOC nursing papers have been published year by year since 2012, and the number of published papers has shown an overall downward trend and has fluctuated up and down before 2021. According to the number of publications, the most influential is “JWOCN,” followed by “Ostomy Wound Management” and “Advances In Skin & Wound Care.”
2. VOSviewer's keyword co-occurrence map analysis yielded eight clusters: (a) prevention, risk factors, skin breakdown, acute care, intensive care, pressure injury (red); (b) ostomy, colorectal cancer, colostomy, peristomal complication, quality of life, self-care, adaption (green); (c) management, wounds care, diabetic food, venous leg ulcers (dark blue); (d) urinary, urinary incontinence, urge incontinence (yellow); (e) pressure, moisture-associated skin damage, barrier, barrier function, skin care (purple); (f) negative pressure wound therapy, device, technology (light blue); (g) care, nursing care, knowledge, nursing education (orange), and (h) telehealth, telemedicine (brown). CiteSpace's keyword time zone map and burst analysis show that wound care has always occupied the hot center of WOC nursing, and pressure injury care is the hot spot among the hot spots. The focus of WOC nursing has shifted from single WOC care to more complex holistic care, such as incontinence-associated dermatitis.
3. The core authors of WOC nursing account for 1.58% (100 in total). The largest connection consisted of 63 projects. Gray Mikel, who has published the most papers, is involved in all three research directions, but his specialty is continence. This is followed by Brett Dave, who mainly focuses on new dressings and management devices. Pittman Joyce, posted third, is also involved in all three directions, but the focus is on the care of pressure injuries.
4. The cooperation between scientific research institutions is not close, with many scientific research institutions having little to no contact with others. UVA, which has the largest number of publications, has published 61 papers related to WOC nursing and has established the closest cooperative relationships with Duke University and the University of Minnesota System, respectively. At the same time, Hollister Incorporated, which ranks second in terms of published articles, and University of Chicago, which ranks third, have also established a stable cooperative relationship. In addition, the overlap rate between institutions with more than 20 publications and the top 15 cited institutions is relatively high. Among them, the 8 American institutions with more than 20 publications are all among the top 15 cited institutions.
5. The United States currently occupies a dominant and authoritative position in this field, followed by Canada, China, and the United Kingdom. The United States and Canada have the closest cooperative relationship, followed by the United States and the United Kingdom. While China ranks third in terms of publications, it is lower in terms of citations and total links. The field of WOC nursing mainly covers the Eurasian continent, and although there are cooperative relationships in scientific research within each continent, the linkages are relatively weak. The WOC nursing field mainly covers the Eurasian continent, and the connections between countries and between continents are mainly maintained by a few countries with a high volume of publications.

Our study uses VOSviewer and CiteSpace to analyze the big data of WOC nursing in the core collection of Web of Science, which can clearly and effectively obtain visual pictures of keywords, co-authors, scientific research institutions, countries, etc. And through these picture information, we can visually understand the current research hot spots and future trends in the field of WOC nursing. The conclusions of our research are as follows: first, scientific research attention in the field of WOC nursing is not high, which is mainly reflected in the fact that the annual number of publications in this field is not high and there is a slight downward trend overall. This may be due to the fact that the current proportion of WOC specialist nurses who have undergone standardized training is not large, and most of them are clinical nurses who are good at practical nursing rather than research-oriented nurses who specialize in scientific research, resulting in an imbalance between scientific research development and practical development in this field. Second, the uneven development of WOC nursing is mainly reflected in the imbalance in the number of publications between journals and countries. For example, only one journal, JWOCN, accounts for 95.57% of the total number of documents. At the same time, there are large differences in documents between the United States and other countries, indicating that there is also an imbalance in the development of WOC nursing between countries. This may be due to the different publication focuses of the magazines and the disparities in the development levels of the countries. Finally, the research focus of WOC nursing is in the wound care sector, among which pressure injuries have always been a research hotspot. And the future development trend of WOC nursing is mainly holistic nursing. The challenge of WOC specialist nurses is to provide comprehensive and professional nursing solutions for patients with complex WOC issues (such as incontinence-associated dermatitis), taking into account practical nursing while promoting the development of WOC nursing research.

To sum up, WOC nursing will require more specialist nurses proficient in both clinical practice and research in the future. And we can promote faster and better development in this field by combining practical nurses and research-oriented nurses. Meantime, cooperation between countries and between scientific research institutions should be strengthened to promote the balanced development of WOC nursing, which can be done through exchanges and learning and scientific research cooperation. In addition, given the breadth and complexity of the fields involved in WOC nursing, medical professional knowledge alone cannot fully solve the problems encountered in practice, such as the improvement and upgrading of new dressings. Therefore, it is necessary to strengthen interdisciplinary cooperation in the future, such as medicine and materials science, medicine and chemistry, and medicine and physics, to help better solve problems and promote development. In recent years, with the rapid development of electronic information, electronic telehealth and telemedicine have provided people with more and more conveniences. In the future, modern electronic technology can also be combined to combine home medical care with telehealth to provide better quality services and convenient WOC nursing to patients at home. Finally, WOC nursing is a comprehensive field, and the balanced development of each sector requires equal attention to each sector, equal attention should be paid to the care of WOC.

## Figures and Tables

**Table 1:** Information about the top 13 journals in the output of WOC nursing papers.

Number	Journals	JCR partition	IF	Number of published papers	%/3474
1	Journal of wound, ostomy, and continence nursing	Q3	1.633	3320	95.57
2	Ostomy wound management	/	/	18	0.52
3	Advances in skin and wound care	Q4	1.600	15	0.43
4	Wound repair and regeneration	Q3	3.560	6	0.17
5	International wound journal	Q3	2.659	6	0.17
6	Journal of tissue viability	Q3	2.279	6	0.17
7	Wound management & prevention	Q4	0.849	6	0.17
8	Journal of wound care	Q4	1.360	4	0.12
9	Psycho-oncology	Q2	3.165	3	0.09
10	AIDS patient care and STDS	Q2	3.073	3	0.09
11	Diseases of the colon and rectum	Q2	2.693	3	0.09
12	Critical care nurse	Q4	1.667	3	0.09
13	Journal of nursing administration	Q4	1.557	3	0.09

**Table 2:** Keyword frequency sorting.

Keyword	Word frequency	Total correlation strength
Ostomy	144	910
Prevention	127	616
Quality of life	125	818
Care	119	672
Prevalence	114	699
Pressure injury	111	605
Pressure ulcer	109	576
Stoma	100	659
Management	91	453
Ulcers	85	370
Colostomy	82	622
Impact	74	501

**Table 3:** Keyword emergence of WOC nursing in 2012–2024.

Keywords	Year	Strength	Begin	End	2012–2024
Women	2012	4.69	2012	2016	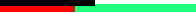

Trial	2012	4.03	2012	2015	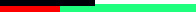

Pressure ulcers	2012	3.29	2012	2015	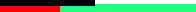

Pressure	2013	3.87	2013	2014	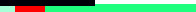

Negative pressure wound therapy	2013	2.87	2013	2014	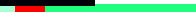

Veterans	2013	2.76	2013	2014	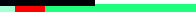

Vacuum-assisted closure	2013	2.76	2013	2014	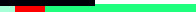

Pressure ulcer prevention	2014	3.87	2014	2015	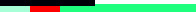

Therapy	2012	3.59	2014	2015	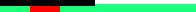

Validity	2014	3.23	2014	2016	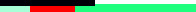

Consensus	2015	3.46	2015	2017	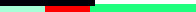

Long-term care	2015	2.67	2015	2016	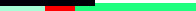

Urinary diversion	2015	2.67	2015	2016	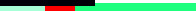

Pressure injury	2015	5.39	2017	2020	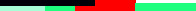

Ulcers	2012	4.41	2017	2018	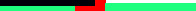

Ulcer prevention	2017	4.34	2017	2019	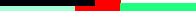

Incontinence-associated dermatitis	2014	4.78	2018	2019	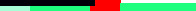

Ulcer risk	2018	2.94	2018	2020	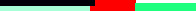

Children	2016	2.69	2018	2019	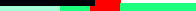

Scoping review	2020	2.64	2020	2021	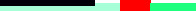

Men	2022	2.98	2022	2024	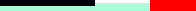

Enhanced recovery	2022	2.98	2022	2024	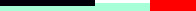

**Table 4:** Author publication statistics.

Author	Number of publications (*n*)	Total connection strength
Gray, Mikel	119	883
Brett, Dave	54	0
Pittman, Joyce	38	380
Chakravarthy, Debashish	35	18
Milne, Catherine T.	35	19
Colwell, Janice	34	396
Bliss, Donna Z.	33	394
Leblanc, Kimberly	26	238
Beeson, Terrie	25	141
Mcnichol, Laurie	25	1080
Ratliff, Catherine R.	24	200
Beitz, Janice M.	21	88
Nichols, Thomas	21	16

**Table 5:** Ranking of research institutions by papers' number.

Organization	Documents	Number of citations	Total connection strength
Univ Virginia	61	993	64
Hollister Inc	43	576	35
Univ Chicago	42	445	60
Univ Minnesota	37	597	26
Med univ South Carolina	35	307	18
Smith & Nephew	30	0	0
Univ Sao Paulo	30	139	13
Rutgers State Univ	29	220	16
Univ Pittsburgh	29	192	19
Univ Tokyo	22	168	9
Duke Univ	21	302	27

**Table 6:** Ranking of research institutions by number of citations.

Organization	Documents	Number of citations	Total connection strength
Univ Virginia	61	993	64
Univ Chicago	42	445	60
Univ Penn	17	210	36
Hollister Inc	43	576	35
Univ Minnesota	37	597	26
Duke univ	21	302	26
Univ Michigan	9	165	24
Indiana univ	15	154	23
Univ Arizona	10	183	22
Yale univ	10	151	22
Indiana univ hlth	17	177	21
Univ Pittsburgh	29	192	19
Med univ, South Carolina	35	307	18
Rutgers state univ	29	220	16
Childrens' hospital, Philadelphia	8	118	15

**Table 7:** Cooperation between countries and total link strength.

Country	Documents	Citations	Total connection strength
United States	1971	8239	164
Canada	170	1297	112
China	99	594	19
United Kingdom	85	1344	111
Brazil	80	439	40
Australia	55	930	82
Japan	50	586	15
Turkey	45	406	18
South Korea	40	235	6
Sweden	30	738	66

## Data Availability

The original contributions presented in the study are included within the article/Supporting Information. Further inquiries can be directed to the corresponding author.
